# Crosstalk Among Circadian Rhythm, Obesity and Allergy

**DOI:** 10.3390/ijms21051884

**Published:** 2020-03-10

**Authors:** Kanami Orihara, Atsushi Haraguchi, Shigenobu Shibata

**Affiliations:** 1 Laboratory of Physiology and Pharmacology, School of Advanced Science and Engineering, Waseda University, Shinjuku-ku, Tokyo 162-8480, Japan; orihara.k.ab@m.titech.ac.jp (K.O.); hmar.h@fuji.waseda.jp (A.H.); 2 School of Life Science and Technology, Tokyo Institute of Technology, Yokohama 226-8501, Japan

**Keywords:** circadian clock system, obesity, metabolic system, immune system, allergy

## Abstract

The circadian clock system works not only as a cellular time-keeper but also as a coordinator for almost all physiological functions essential to maintaining human health. Therefore, disruptions or malfunctions of this system can cause many diseases and pre-symptomatic conditions. Indeed, previous studies have indicated that disrupted clock gene expression rhythm is closely related to obesity, and that allergic diseases can be regulated by controlling peripheral clocks in organs and tissues. Moreover, recent studies have found that obesity can lead to immune disorders. Accordingly, in this review, we assess the connection between obesity and allergy from the point of view of the circadian clock system anew and summarize the relationships among the circadian clock system, obesity, and allergy.

## 1. Introduction

Almost all plants and animals live in response to 24-hour cycles of the light-dark environment generated by the rotation of the earth. However, even in an environment that has no light-dark changes and no time information, we live under an approximately 24-hour cycle. This rhythm is known as a circadian rhythm. The circadian clock system generates the circadian rhythm and modulates the sleep-wake cycle, body temperature fluctuation, neural activity, and hormone secretion rhythm, all of which enable the body to function in response to a 24-hour cycle [1].

The circadian clock system consists of transcriptional-translational negative feedback loops between clock genes. *Period1/2* (*Per1/2*), *Cryptochrome1/2* (*Cry1/2*), *Bmal1* (*Brain and muscle arnt-like protein 1*), and *Clock* (*Circadian locomotor output cycles kaput*) are the main componential genes of the core loop involved in the creation of an approximately 24-hour period. CLOCK heterodimerizes with BMAL1, and CLOCK:BMAL1 heterodimers act as transcriptional promoters for *Per* and *Cry* through binding with the specific promoter sequence known as the E-box. *Per* and *Cry* mRNAs then translocate from the nucleus to the cytoplasm and are translated into PER and CRY proteins. PER and CRY form a heterodimer and return to the cytoplasm. PER:CRY heterodimers then act to inhibit *Per* and *Cry* transcription through binding to CLOCK:BMAL1 heterodimers that bind with the E-box. As a consequence, *Per* and *Cry* mRNA and protein levels show circadian changes [2]. Moreover, other feedback loops exist alongside this core loop. In another loop, *Retinoic acid receptor-related orphan receptor* (*Ror*) and *Reverse erythroblastosis virus* (*Rev-erb*) are regulated by the core loop, and these proteins enhance and inhibit transcription of *Bmal1*, respectively, through binding to another specific ROR response element (RORE). Moreover, *Rev-erb* inhibits transcription of *Clock* through binding with the REV-ERB response element (RevRF). In addition to these loops, post-translational modifications, such as phosphorylation, ubiquitination, and subcellular trafficking, contribute to the maintenance of the circadian clock system by modulating the stability of the clock gene proteins [3].

In mammals, almost all cells of the body share the molecular mechanism described above. These clock gene systems are divided into central and peripheral clocks, depending on the organs in which they exist [4]. The central clock exists in the suprachiasmatic nucleus (SCN) of the hypothalamus, while peripheral clocks exist in almost all peripheral organs and brain regions other than the SCN. The central and peripheral clocks form a hierarchical system. In general, the central clock is entrained by environmental light, through the retinal-hypothalamic tract, for eliminating the difference between the environmental clock and the circadian clock. Subsequently, the central clock activates neural signals, hormonal signals, locomotor activity, and other pathways to adjust the peripheral clocks to the environmental clock [5]. Accordingly, environmental light is an essential factor for keeping the biological rhythm at 24 h, because the period length created by the circadian clock system is longer than 24 h. Indeed, the period length of the sleep-wake cycle was shown to be 24.5–25 h for participants living without sunlight and time information [6]. Moreover, previous studies showed that SCN-lesioned mice or mice under light-light (LL) conditions, where the central clock is malfunctioning, showed arrhythmic locomotor activity and feeding rhythm. Besides, peripheral clocks in these mice showed different phases for each organ, and their amplitudes were lower than those in mice kept under usual light-dark (LD) conditions [7,8]. Taken together, these studies show that the central clock orchestrates other biological clocks and that environmental light is the only entrainment factor of the central clock.

While the central clock is entrained only by environmental light, peripheral clocks can be entrained by many stimuli, such as temperature, meal, exercise, and stress [9]. A previous study showed that delaying the timing of three meals a day (breakfast, lunch, and dinner) by 5-hours delayed the clock gene expression rhythm in adipose tissue in humans. This study also showed that the secretion rhythms of cortisol and melatonin, which are biomarkers for evaluating the rhythm of the central clock, remained unaffected [10]. Another study, which used RNA from the hair follicle cells in humans, demonstrated that nighttime exercise (from 20:00 to 22:00) delayed the phase of clock gene expression rhythm for 2 to 4 h, as compared to that without exercise [11]. Studies in mice have shown that many environmental factors, such as restricted feeding (RF) during the inactive period, wheel-running exercise only during the beginning or end of the active period, and physical and psychological stress, affected the phase of peripheral clocks, but not the central clock [12,13]. Moreover, these studies in mice identified insulin and cortisol as the main entrainment factors when the peripheral clocks are affected by feeding, exercise, and stress [12,13]. Therefore, whereas the central clock is entrained only by the environmental light, the peripheral clocks are orchestrated by the central clock but also affected by external stimuli, including feeding, exercise, and stress.

In the circadian clock system, the clock genes regulate not only expression rhythm of themselves, but also the expression rhythm of clock-controlled genes (CCGs). The CCGs are involved in many physiological functions, such as metabolism, immunity, and other functions. Indeed, previous studies showed that CCGs represent approximately 10% of all genes in many organs [14]. Consequently, studies in humans showed that postprandial blood glucose levels in the evening are maintained at a higher level than in the morning [15]. Moreover, an additional study demonstrated that a late dinner increases the maximum of postprandial blood glucose levels compared with an early dinner [16]. In addition, circadian rhythms have been observed in the occurrence of various diseases, such as asthma, myocardial infarction, and depressive symptoms, due to the circadian rhythm of hormone secretion, neural activity, and other physiological functions [17]. Indeed, studies in mice revealed that glucose tolerance testing at the beginning or middle of the active period produced lower blood glucose levels than the idle period [18]. Other studies showed that food antigen exposure in the late inactive period caused more severe food allergy symptoms than in the late active period [19] and that salivary IgA secretion rhythm was abolished by SCN lesion [20]. Therefore, keeping an accurate circadian clock system is vital for maintaining normal physiological functions.

In this review, we limit discussion to the mammalian circadian clock system and focus on the interplay among the circadian clock system, obesity, and allergy. Moreover, we discuss not only phenomenological studies in humans but also fundamental mechanisms using mice and other animal experiments. In doing so, we hope this review improves the understanding of these interactions.

## 2. Circadian Rhythm in Obesity

The increase in the number of people with obesity is a global public health concern, which poses a substantial socioeconomic burden. Furthermore, epidemiologic studies have indicated that obesity increases the risk of diabetes, insulin resistance, metabolic syndrome, and cardiovascular disease [21,22]. Many studies in humans have shown that obesity associates with disturbances of biological rhythms, such as those governing sleep and food intake. Additionally, studies have shown that people with obesity have delayed bedtimes and shorter sleep duration compared to healthy people. In one study, plasma melatonin circadian rhythm in people with obesity was disrupted, and no significant circadian rhythm was found [23]. In another study of mainly overweight women, there was a negative correlation BMI with urinary 6-sulfatoxymelatonin concentration at 09:00, which is a metabolite of melatonin and shows rhythm similar to melatonin secretory rhythm [24], suggesting that overweight women’s melatonin rhythm might be disturbed. The reason is that serum melatonin and urinary 6-sulfatoxymelatonin show similar secretory rhythm. Moreover, nocturnal hyperphagia and morning anorexia, which were regarded as delayed food intake pattern, was observed in a group of obese women, which is characteristic of night eating syndrome [25]. In brief, obesity is involved in not only the malfunction of the metabolic system but also the disturbance of biological rhythms.

On the other hand, obesity is caused by a disrupted lifestyle rhythm, such as shift work and varying meal timings. In terms of shift work, previous human epidemiological studies indicated that shift work, especially the duration of shift work, is associated with an increased risk of obesity [26,27]. Regarding the timing of meals, studies showed that skipping the first meal of the day (breakfast) increases the risk of obesity [28,29], that the multivariable-adjusted odds ratio of obesity was even higher with a late dinner and bedtime snack than with skipping breakfast [30], and that subjects given 2000 calories in a single daily meal for a week showed greater body weight loss when this meal was given at breakfast rather than at dinner [31]. Shift work and varying meal timings not only are associated with the increased risk of obesity but also alter the biological rhythm. Previous studies have shown that nocturnal light exposure increased the secretion of insulin and GLP-1 after meals [32] and that the acute stimulation of night work for 4 days slowed down the increase in postprandial blood glucose levels and reduced insulin sensitivity [33]. Another study suggested that a positive correlation between body fat percentage and the percentage of calories consumed 4 h before dim light melatonin onset (DLMO; a biomarker for the beginning of the biological night) or sleep onset [34]. From these papers, a late food intake corresponded to the biological clock, independent of amount or content of food intake and activity level, would be associated with increased body weight and/or obesity. In addition to these effects of shift work and varying meal timings on metabolic parameter, Indeed, previous studies in humans showed that the shift work shifted the phase of clock gene expression rhythm and the levels and timing of melatonin production [35,36], and that delayed three meals (breakfast, lunch, and dinner) for 5-hour delayed clock gene expression rhythms in adipose tissue [10]. Taken together, evidence suggests that a disrupted lifestyle rhythm, such as that seen with shift work and varying meal timings, is a contributing factor to obesity, and obesity interacts closely with a disrupted lifestyle rhythm.

These interactions have also been observed in many studies in mice and rats. There are many rodent models of obesity and diabetes, such as ob/ob mice and db/db mice, which are deficient in leptin and the leptin receptor, respectively, KK-*A^y^* mice, which are obese and diabetic mice independent of insulin, and high-fat diet (HFD) induced obesity (DIO; diet-induced obesity) model mice. Many previous studies have indicated that these obesity model mice show disruption of biological rhythms, such as locomotor activity rhythm, food intake rhythm, and clock gene expression rhythms. For example, obese ob/ob mice show a phase shift and decreased rhythmicity of the sleep-wake cycle and locomotor activity rhythm [37,38], and the amount and amplitude of their locomotor activity and energy expenditure are decreased significantly compared with healthy mice [39]. Further, diabetic obese db/db mice show no circadian rhythmicity in locomotor activity, and their REM duration in the inactive period is decreased, although their clock gene expression rhythm in central and peripheral clocks shows circadian rhythmicity [40,41]. Moreover, the changes in metabolic parameters, such as plasma glucose, insulin, liver glycogen, and hepatic glycogen synthase and phosphorylase activities, in diabetic obese db/db mice may be caused by changes in daily rhythm rather than by absolute changes [42]. Also, obese and diabetic KK-*A^y^* and DIO mice show attenuated expression rhythm of clock genes and downstream target genes of clock genes in the liver and central nervous system [43,44,45,46]. Thus, these studies indicate that obese mice, regardless of model, show disruptions of biological rhythm and that some types of obese model mice dysregulate their clock gene and CCG expression rhythms.

Contrarily, other studies have indicated that disrupted biological rhythm, caused by altered light conditions, HFD, or clock gene deficiency, increases the risk of obesity. In rats, the chronic advance of the light period increased appetite, and decreased metabolism and energy expenditure, while promoting significant alterations in neuropeptides, lipid metabolism, and inflammation [46]. Moreover, feeding mice with an HFD increased food intake during the inactive period and decreased food intake during the active period, leading to a reduction in food intake rhythmicity. These effects are caused by activated microglia that promote hypothalamic inflammation [47]. Besides, this diet decreased locomotor activity during the day and reduced the synchronization to light [48,49]. Previous studies have also suggested that the effects of HFD on biological rhythm are due to disturbed clock gene and CCG expression rhythm, which may be caused by disrupted food intake rhythm. Indeed, it has been demonstrated that feeding with HFD shifts the phase of clock gene and CCG expression rhythm in many peripheral organs, disturbs the phosphorylation rhythms of the metabolic regulators CREB and S6, impairs CLOCK:BMAL1 recruitment, and promotes activation of pathways through the transcriptional regulator PPARγ [50,51,52]. On the other hand, several papers suggest that mutations or deletions of clock genes are related to dysfunctional energy metabolism. *Cry* mutation mice show dysfunctions in insulin secretion and hyperglycemia [53]. In *Bmal1*-KO (knockout) mice, disorders of lipid metabolism, independent of food components, a high respiratory quotient, which indicates difficulty utilizing lipids as an energy source, and lower insulin secretion are seen compared to wild-type mice [54,55]. Moreover, studies of these mice have indicated that PEPCK (phosphoenolpyruvate carboxykinase) and GLUT2 (glucose transporter 2), which are involved in glucose metabolism, showed low expression levels and arrhythmic expression [56]. In *Clock* mutant mice, arrhythmicity of food intake and dysfunction of the metabolic system are seen [57]. Moreover, these mice show severe hypoglycemia after insulin injection due to impaired gluconeogenesis [58]. Liver-specific *Rev-erb*α and β double KO mice show arrhythmic expression of *Ppar*α, which is involved in β-oxidation of fatty acids [59]. Thus, disruption of the circadian clock system, caused by light conditions, HFD, and gene deletion, interacts with impairment of the metabolic system, leading to obesity.

## 3. Obesity and Allergy

Obesity reportedly causes an increase in the levels of inflammatory mediators, potentially leading to immune disorders. In the obese state, the levels of plasma adiponectin, an anti-inflammatory adipokine, are low, thus enabling the natural onset of inflammation [60]. Leptin and adiponectin are adipokines secreted by white adipose tissue and are known to be related to obesity and involved in glucose and lipid metabolism. Leptin, a hunger-inhibiting hormone predominantly made by adipocytes, is a mitogen factor for keratinocytes that also promotes fibroblast proliferation and positively correlates with body fat and body fat mass [60,61]. A study conducted in the United States of America revealed a positive correlation between BMI and total IgE levels in children aged 2 to 19 years old [60]. Interestingly, this study showed a relationship between CRP and total IgE levels with age-adjustment, which was also confounded by BMI [60]. Since obesity is associated with atopy, reflecting an inflammatory state, it may be correlated with food allergies [62]. Although it is a rather small study, one of the recent cohort studies with 164 children showed that obesity in girls at 2 years of was highly correlated with increased risk of asthma, with odds ratio 12.14 [63]. In adult study, 9,888 Japanese subjects study reported that obese significantly increases a risk of late-onset asthma only in women [64]. Meta-analysis of 18 children-subjected articles revealed that overweight or obese showed 1.30-fold increase in a risk of childhood asthma, and 1.90 for wheeze [65]. Another meta-analysis with 13 studies showed a positive correlation with 1.47 odds ratio between abdominal obesity and asthma [66]. Taken together, these studies have suggested that a higher prevalence of asthma is observed among obese and overweight adults and children, who are also at risk of increased severity and a worse prognosis [39,63,64,65,66,67]. However, precise mechanism of obesity causing asthma in children are still not clear, while late-onset asthma is mostly neutrophil-predominant and non-IgE-mediated, which shows increased levels in adipokines [68]. Moreover, a body mass index exceeding 30 kg/m^2^ is associated with a 92% increased risk of developing asthma [69]. Furthermore, gastric bypass surgery was shown to significantly improve airway hyperresponsiveness in obese individuals with asthma who had normal serum IgE levels, but not in those with elevated IgE [70]. Several large-scale studies have reported that obesity positively correlated with allergic rhinitis (AR) and chronic rhinosinusitis (CRS) in both adults and children [61]. Additionally, a Spanish study showed a positive relationship between BMI and the severity of atopic dermatitis (AD) [66]. Further, although within the standard range, total cholesterol levels in patients with AD were higher than those in a healthy group, and were found to be yet higher in cases of severe AD [71]. Although skin dryness is one of the risk factors for AD, contrasting results have been reported regarding transepidermal water loss (TEWL) and obesity. A significant positive correlation was reported between BMI and TEWL [72]. Additionally, increased TEWL was observed in obese children [73], while one study showed decreased TEWL in obese individuals [74]. The authors attributed this result to the role of adipokines and leptin [74].

So far, we have shown evidence of the positive correlation between obesity and allergic diseases. Interestingly, a study showed that the prevalence of AR/CRS and AD was higher in obese individuals than in those with asthma and food allergies [61]. These phenomena can be partially explained by interleukin (IL)-17 involvement. Anti-IL-17 monoclonal antibody treatment (Secukinumab) is an available option for the treatment of psoriasis, and it has shown a better, more rapid response in patients with a healthy BMI than overweight (BMI ≥ 25) patients [75]. This result suggests that IL-17 neutralization is less effective in obese patients because of obesity-mediated inflammation. Additionally, allergic diseases with higher involvement of type 3 inflammation (IL-17-related response) may show a stronger correlation with obesity. This observation is supported by the failure to induce obesity in IL-17 receptor A (IL-17RA) knockout mice [76].

In animal studies, obese mice were shown to develop allergic sensitization and severe airway eosinophilia with a smaller amount of allergen compared to lean mice. Obesity also decreased the threshold of allergic sensitization, as smaller amounts of allergen were sufficient to induce a comparable level of allergen-specific antibodies, as compared with lean mice [67]. Another study showed that HFD significantly increased mast cell accumulation in the intestine and enhanced intestinal permeability [77]. Interestingly, both of these effects are promoted by the induction of food allergy in these mice [77]. Microbiota transplantation experiments have also shown that HFD-associated microbiota enhanced susceptibility to food allergy, but did so independently of obesity [77]. Interestingly, the allergen-specific IgE levels of HFD-fed animals remained comparable to controls [77]. This finding suggests that IgE induces the increased intestinal mast cell response. Nevertheless, these studies suggest that obesity is a risk factor for developing allergic diseases, although the influence of atopic factors appears to be stronger.

## 4. Circadian Rhythm in Allergy

Recently, a large genome-wide association study identified *RORA* as one of the significant genes associated with asthma [78]. *RORA* encodes a member of the NR1 subfamily of nuclear hormone receptors that binds to ROREs in DNA as a monomer. *Rora* was reported to enhance *Bmal1* transcription and play an essential role in maintaining a robust circadian rhythm [79]. In immunity, RORα and RORγ are well known as key transcription factors for Th17 [80] and type 3 innate lymphoid cell [81] differentiation, both of which are important in chronic allergic diseases. RORα is also an important factor for type 2 innate lymphoid cells (ILC2), and when it was knocked down together with IL-7R, ILC2-deficient mice were obtained [82]. Using this strain, the authors demonstrated a partial decrease of type 2 airway inflammation in an asthma model [82]. Collectively, both type 2 and 3 inflammation are under the control of RORα, which plays a vital role in chronobiology by enhancing Bmal1 transcription. Together with BMAL1, RORα is one of the major regulators of the circadian molecular oscillator. Therefore, the peculiar role of RORα suggests that allergy is one of the responses under the influence of circadian homeostasis.

Recently, Spadaro et al. showed that histamine release from basophils oscillates and significantly fits a cosine curve in asthmatic patients, but not in healthy controls [83]. They also showed a less dramatic amplitude in daily cortisol rhythm in patients with asthma [83], suggesting the involvement of disrupted local molecular clocks in asthmatic physiology. However, a relatively small Korean study reported, interestingly, that CCGs from nasal mucosa in the right and left turbinates showed asymmetric expression levels [84]. The authors also found higher expression levels of these genes in AR patients, compared to healthy controls [84]. This suggests that the local molecular clock is dysregulated or shifted in the lesion site. Although a causality dilemma for this phenomenon is still not fully clarified, resetting the local clock can be one of the options to improve from this condition.

In humans, two single nucleotide polymorphisms (SNPs) of *CLOCK* were reported to enhance the risk of overweight or obesity by 1.8-fold [85]. Another study of *CLOCK* SNPs reported that three SNPs were associated with plasma cytokine levels, especially IL-6, adiponectin, and CCL2/MCP-1 [86]. Separately, variable number tandem repeats (VNTRs), such as 86 bp repeats in IL-1 receptor antagonist (IL-1RA) intron 2 and 70 bp in IL-4 intron 3, were reported to be associated with obesity and adiposity in a Malaysian study [87]. Apolipoprotein ApoA-IV, found on high-density lipoproteins (HDL) cholesterol in serum, as well as in circulation, is known to have anti-inflammatory effects. ApoA-IV was reported to inhibit eosinophil chemotaxis towards CCL11/eotaxin and prostaglandin D2 (PGD2) via an NR1D1 (Rev-erbα)-dependent pathway [88]. The authors also examined the systemic effects of ApoA-IV using an asthmatic murine model, and showed a significant decrease in airway hyperresponsiveness with exogenous ApoA-IV administration [88].

Using mice, Ehlers et al. showed, interestingly, that, compared to controls, *Bmal^−/−^* or chronic jet lag mice developed severe acute viral bronchiolitis in response to Sendai virus and influenza A virus [89]. Similar results were also reported in myeloid cells of mice lacking BMAL1, which developed severe symptoms in an ovalbumin-induced asthmatic model [90]. Additionally, Nakamura et al. showed that a Clock mutation in mast cells caused a defect in FcεRI, the high-affinity IgE receptor, expression, leading to an inadequate IgE-mediated degranulation [91]. They also reported that glucocorticoids inhibited IgE-mediated allergic reactions and increased PER2 levels, leading to a resetting of the local molecular clock in mast cells, both in vitro and in vivo [92]. Speaking of mast cells, circadian oscillations in mast cell degranulation were reported to be attenuated by inhibition of FcεRI [92] or organic cation transporter 3 (OCT3) [93], both of which expression levels are temporarily regulated by Clock in their E-box motif in promoter region. These findings imply that mast cell functions in allergy can be regulated by recalibrating local molecular clocks.

## 5. Conclusions

**Figure 1:**
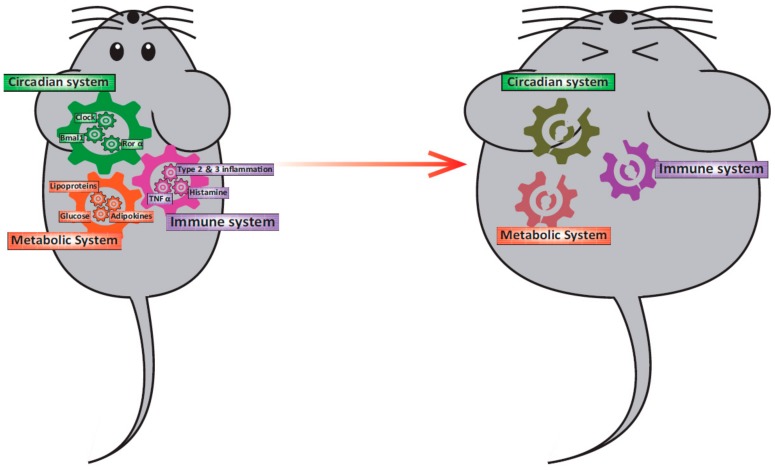
Relationship among the circadian clock system, obesity, and immunity. In a healthy state, the circadian clock system, the metabolic system, and the immune system interact and function normally. However, when one of the gears became out of position for some reason, other systems will be led to malfunction.

Here, we summarize evidence from in vitro, ex vivo, and in vivo studies of rodents and humans, which show the interplay among the circadian clock system, obesity, and allergy (Figure 1).

The reviewed studies suggest that the dysfunction of biological rhythms, irrespectively of the underlying cause, might lead to obesity. However, obesity also triggers arrhythmicity in clock gene expression and biological rhythms. Studies in mice have shown that time-restricted feeding (TRF) with HFD, independent of TRF timing, has an anti-obesity effect. This effect is mediated through the improvement of the disrupted clock gene and CCG expression and energy expenditure rhythms brought about by HFD feeding [50]. A recent study in humans confirmed that participants’ body weight was reduced, and their sleep satisfaction was improved when eating duration, or the time from the first meal (breakfast) to the last meal (dinner or night snack), is restricted to 11 h [94]. Thus, regulating meal timings to correspond to one’s biological rhythm would be helpful to attenuate obesity.

A growing body of evidence has reported on the relationship between circadian rhythm and allergy, but the understanding of the mechanisms underpinning this relationship is still limited. Interestingly, the phenomenon of nocturnal asthma has been discussed since the 1980s [95]. However, the molecular mechanisms by which the circadian system controls the pathophysiology of this condition remain to be elucidated. Recent studies in a variety of fields, including circadian system [96], and allergy [97,98] research, have focused on the microbiota. These reports suggest the importance of studying microbiota, in addition to examining probiotics and prebiotics, which may be key to investigating the unknown molecular mechanisms in these fields.

In this review, we assessed the connection between obesity and allergy from the viewpoint of the circadian system anew. Although we may not cover the whole field of disease phenotypes, we believe this review highlights the intimacy with which these three physiological aspects interact to maintain homeostasis. Further direct evidence to dissect the complex, mutual relationships between allergy and the circadian clock is required and may lead to a more in-depth understanding of the reciprocal interactions among the circadian clock, obesity, and allergy.
